# Assessing THK523 selectivity for tau deposits in Alzheimer’s disease and non–Alzheimer’s disease tauopathies

**DOI:** 10.1186/alzrt240

**Published:** 2014-02-26

**Authors:** Michelle T Fodero-Tavoletti, Shozo Furumoto, Leanne Taylor, Catriona A McLean, Rachel S Mulligan, Ian Birchall, Ryuichi Harada, Colin L Masters, Kazuhiko Yanai, Yukitsuka Kudo, Christopher C Rowe, Nobuyuki Okamura, Victor L Villemagne

**Affiliations:** 1 The Florey Institute of Neuroscience and Mental Health, 30 Royal Parade, Parkville, 3052 Melbourne, Victoria, Australia; 2 Department of Nuclear Medicine & Centre for PET, Austin Health, 145 Studley Road, Heidelberg, 3084 Melbourne, Victoria, Australia; 3 Department Pharmacology, Tohoku University School of Medicine, Sendai, Japan; 4 Department of Anatomical Pathology, The Alfred Hospital, Monash University, Melbourne, Australia; 5 Innovation of New Biomedical Engineering Center, Tohoku University, Sendai, Japan

## Abstract

**Introduction:**

The introduction of tau imaging agents such as ^18^F-THK523 offers new hope for the *in vivo* assessment of tau deposition in tauopathies such as Alzheimer’s disease (AD), where preliminary ^18^F-THK523-PET studies have demonstrated significantly higher cortical retention of ^18^F-THK523 in AD compared to age-matched healthy individuals. In addition to AD, tau imaging with PET may also be of value in assessing non-AD tauopathies, such as corticobasal degeneration (CBD), progressive supranuclear palsy (PSP) and Pick’s disease (PiD).

**Methods:**

To further investigate the ability of THK523 to recognize tau lesions, we undertook immunohistochemical and fluorescence studies in serial brain sections taken from individuals with AD (*n* = 3), CBD (*n* = 2), PSP (*n* = 1), PiD (*n* = 2) and Parkinson’s disease (PD; *n* = 2). In addition to the neuropathological analysis, one PSP patient had undergone a ^18^F-THK523 PET scan 5 months before death.

**Results:**

Although THK523 labelled tau-containing lesions such as neurofibrillary tangles and neuropil threads in the hippocampus and frontal regions of AD brains, it failed to label tau-containing lesions in non-AD tauopathies. Furthermore, though THK523 faintly labelled dense-cored amyloid-β plaques in the AD frontal cortex, it failed to label α-synuclein-containing Lewy bodies in PD brain sections.

**Conclusion:**

The results of this study suggest that ^18^F-THK523 selectively binds to paired helical filament tau in AD brains but does not bind to tau lesions in non-AD tauopathies, or to α-synuclein in PD brains.

## Introduction

Alzheimer’s disease (AD) is the most common form of dementia (50% to 70% of dementia cases) [1]. At present, there is no cure for the disease. Age is the greatest risk factor. Despite the existence of distinctive clinical diagnostic criteria, the differential diagnosis of AD and other neurodegenerative disorders is sometimes challenging because of substantial overlap in clinical presentations, especially at the early stages of the disease [2]. Consequently, making the definitive diagnosis of neurodegenerative diseases is still reliant upon postmortem examination of the brain.

AD is pathologically characterised by the presence of (1) extracellular neuritic plaques composed of aggregated β-amyloid (Aβ) and (2) intracellular neurofibrillary tangles (NFTs) composed of the aggregated tau protein [3,4]. Tau aggregates are a pathological trait of not only AD but also other neurodegenerative conditions, such as corticobasal degeneration (CBD) and progressive supranuclear palsy (PSP), as well as some variants of frontotemporal lobar degeneration (FTLD-tau) [5], such as Pick’s disease (PiD). Whilst the underlying mechanism leading to tau accumulation remains unclear, it is thought to be related to several pathogenic events resulting in hyperphosphorylation, misfolding and aggregation of tau. Tau aggregation in this wide spectrum of tauopathies presents with different morphologies (for example, NFTs in AD, astrocytes in CBD, globose tangles and thorny and tufted astrocytes in PSP and Pick bodies in PiD [6-9]) and ultrastructural conformations (for example, paired helical filaments in AD, straight filaments in PSP and twisted ribbons and random coils in PiD [6,10,11]), which are probably attributable to the combinations of the different tau isoforms and a wide variety of posttranslation modifications [6,12]. Additionally, the spatial distribution of the tau aggregates in these tauopathies differ from each other, with NFTs in AD being prevalent in the mesial temporal cortex and cortical grey-matter (GM) areas. Tau aggregates are also found in the frontal and striatal brain regions in CBD; in the brainstem, cerebellar white matter and basal ganglia in PSP; and in the frontal and temporal neocortex in PiD [13-17]. The diverse distribution of these tau aggregates in the brain can potentially be useful in the differential diagnosis of these tauopathies, assuming the same tau imaging agent binds with similar affinity to the whole spectrum of tau aggregates. Alternatively, the differential diagnosis might require the development of selective tau radiotracers for each specific conformation of tau aggregates.

In recent years, a great deal of interest has been placed on identifying the ideal diagnostic tool for neurodegenerative diseases. Despite the quantitative assessment of Aβ, tau and phospho-tau in cerebrospinal fluid (CSF) [18], lumbar puncture is still considered an invasive procedure for the widespread screening of the ‘at-risk’ population. Additionally, CSF measurements do not provide information on regional brain deposition of Aβ or tau, which may have clear correlates with cognition or regional brain atrophy and might not be able to provide important information regarding the therapeutic outcomes or response to current drugs aimed at modulating the deposition of these misfolded proteins [19-23]. Given the sometimes nonspecific nature of clinical symptoms and neuropsychological assessments, modern molecular imaging techniques have proven beneficial in the noninvasive identification of the underlying pathology of these diseases. Considerable effort has been focused on the development of novel Aβ ligands that permit detection of Aβ deposition [24]. The Aβ-specific ligands ^18^F-AV-45 (florbetapir; (*E*)-4-(2-(6-(2-(2-(2-([^18^F]-fluoroethoxy)ethoxy)ethoxy)pyridin-3-yl)vinyl)-N-methyl benzenamine) and Pittsburgh compound B (PiB) [25] are the best characterized and have proven to be suitable positron emission tomography (PET) biomarkers for the *in vivo* quantitation of cerebral Aβ burden. They have demonstrated a robust difference in retention between AD and healthy individuals [25-27]. ^18^F-AV-45 [27] and flutemetamol 18 (2-[3-fluoranyl-4-(methylamino)phenyl]-1,3-benzothiazol-6-ol) [28] have already been approved for clinical Aβ imaging in the United States. These two agents belong to a second generation of Aβ radiotracers labelled with ^18^F, which, with a half-life of 110 minutes, allows a wider and more cost-effective application of Aβ imaging.

We recently reported the preclinical characterization of the selective tau radiotracer ^18^F-THK523 [29], a quinoline derivative pioneered by Okamura and colleagues [30,31]. Preliminary clinical evaluation of ^18^F-THK523 has demonstrated that ^18^F-THK523 retention is significantly higher in the cortical and hippocampal GM of AD patients than in age-matched healthy individuals [32].

To discern whether ^18^F-THK523 recognises non-AD tau aggregates in addition to NFTs, we evaluated a series of brain sections from AD and non-AD tauopathies to evaluate the binding profile of ^18^F-THK523.

## Methods

### Postmortem assessment

#### Chemicals

All reagents were purchased from Sigma-Aldrich (St Louis, MO, USA) unless otherwise stated.

#### Tissue collection and characterisation

Tissues were sourced and prepared by the Victorian Brain Bank Network. The AD pathological diagnosis was made according to standard National Institute on Aging/Reagan Institute criteria [5]. Determination of age-matched control cases were subject to the above-described criteria. The pathological diagnoses of PiD, CBD and PSP were all made according to previously described methods [33,34]. Ten cases were evaluated for this study: AD (*n* = 3), CBD (*n* = 1), PiD (*n* = 2), PD (*n* = 1) and PSP (*n* = 3). One of the individuals with PSP had undergone ^18^F-THK523 PET 5 months before death.

#### Immunohistochemistry and fluorescence analysis

All brain tissue was fixed in 10% neutral buffered formalin, processed, and embedded in paraffin. For immunohistochemistry, 5-μm serial sections were deparaffinized and treated with 90% formic acid for 5 minutes, and endogenous peroxidase activity was blocked with 5% hydrogen peroxide. Sections were then treated with 0.2% casein in Tris buffer before incubation with primary antibodies to α-synuclein (97/8, 1:2,000 dilution) [35], Aβ (1e8, 1:2,000 dilution; monoclonal antibody recognises Aβ(17–24)) [36] and tau (polyclonal antibody recognises C-terminal tau (amino acids 243 to 441), catalog no. 0024; Dako Denmark, Glostrup, Denmark), for 1 hour at room temperature. Serial 5-μm tissue sections were stained as follows. The first and third sections were immunolabelled with anti-97/8 antibody, anti-1e8 antibody or tau to identify Lewy bodies, Aβ plaques or tau aggregates, respectively. The second serial section was stained with unlabelled THK523 to assess whether THK523 staining colocalised with the immunodetected Lewy bodies and/or Aβ plaques and/or tau aggregates. Detection of antibody binding was achieved using the LSAB kit (labelled streptavidin-biotin, catalog no. K0657; Dako Denmark), then sections were incubated with hydrogen peroxidase diaminobenzidine (H_2_O_2_-DAB; Dako Denmark) to visualise the α-synuclein-, Aβ- or tau-positive deposits. Sections were counterstained briefly (15 seconds) with Harris’s haematoxylin. To detect THK523 fluorescence, quenching was first performed whereby sections were first deparaffinized and tissue autofluorescence was minimized by treatment of sections with 0.25% KMnO_4_ phosphate-buffered saline (PBS) for 20 minutes prior to washing in PBS and incubation with 1% potassium metabisulphite/1% oxalic acid/PBS for 5 minutes. Following autofluorescence quenching, sections were blocked in 2% bovine serum albumin/PBS, pH 7.0, for 10 minutes and stained with 100 μM THK523 for 30 minutes. Sections washed in PBS were then mounted in nonfluorescent mounting medium (catalog no. S3023; Dako Denmark). Epifluorescent images were visualized on a Leica microscope (47-nm cyan fluorescent protein, fluorescence filter set 47 (EM BP 436/20, BS FT 455 and EM BP 480/40); Leica Microsystems, North Ryde, 2113 Australia). Colocalisation of the THK523 and antibody signals were assessed by overlaying images from each of the stained serial tissue sections.

### Antemortem assessment

Five months before death, a seventy-nine-year-old patient diagnosed with PSP underwent an Aβ imaging PET scan with ^18^F-florbetaben and a tau imaging scan with ^18^F-THK523. Approval of the study was granted by the Austin Health Human Research Ethics Committee, and written informed consent was obtained from all participants and caregivers before the study. The patient was recruited, reviewed and diagnosed on the basis of clinical and neuropsychological assessment by consensus of a neurologist and a neuropsychologist.

As part of the imaging protocol, we performed magnetic resonance imaging (MRI) using a three-dimensional magnetization-prepared rapid acquisition gradient echo sequence and T2-weighted fast spin echo and fluid-attenuated inversion recovery sequences. Both ^18^F-florbetaben and ^18^F-THK523 were synthesized at the Centre for PET, Austin Health, as previously described [37-39]. PET scans were acquired using a Philips Allegro PET scanner (Philips Healthcare, North Ryde, Australia) at the Austin Health Centre. A transmission scan using a rotating Cs-137 source was taken for attenuation correction immediately prior to obtaining the emission scan. A 60-minute list-mode emission acquisition, followed by a 90- to 120-minute acquisition using 10-minute frames, was performed in three-dimensional mode after injection of 300 MBq of ^18^F-florbetaben. A 90-minute list-mode emission image acquisition was performed in three-dimensional mode after injection of 200 MBq of ^18^F-THK523. Images were reconstructed using a three-dimensional row action maximum likelihood algorithm.

PET images were processed using a previously described semiautomatic region of interest (ROI) method [40]. Briefly, coregistration of the patient’s MRI scans with the PET images was performed with Statistical Parametric Mapping 8 (SPM8) software [41]. A narrow cortical ROI template was placed on the coregistered MRI scanner by an operator (VLV) who was blinded to the participant’s clinical status, then it was transferred to the coregistered PET images. The ROI template covered cortical and subcortical GM structures as well as the midbrain and pons. Subcortical white-matter ROIs were placed at the centrum semiovale, and the cerebellar regions were placed over the cerebellar cortex, taking care to avoid white matter. Standardised uptake values (SUVs), defined as the decay-corrected brain radioactivity concentration normalized for injected dose and body weight, were calculated for all regions. In order to avoid arterial blood sampling, a simplified approach was applied using the cerebellar cortex as the reference region. SUVs were used to derive SUV ratios (SUVRs) referenced to the cerebellar cortex soon after the ratio of binding in neocortex to that in the cerebellar cortex reached an apparent steady state. Regional THK523 SUVRs were obtained for all regions sampled. Global tau burden was expressed as the average THK523 SUVR for the following cortical ROIs: frontal (consisting of the dorsolateral prefrontal, ventrolateral prefrontal and orbitofrontal regions), superior parietal, lateral temporal, lateral occipital, and anterior and posterior cingulate. Partial volume correction accounting for both GM atrophy and white-matter spillover was performed using a three-compartment approach with PMOD version 3.1 software (PMOD Technologies, Zurich, Switzerland). To establish whether either ^18^F-florbetaben or ^18^F-THK523 retention in the PSP patient was different from age-matched controls, a Z-score was generated for both global and regional retention. The respective Z-scores were generated against ten healthy controls who had ^18^F-florbetaben studies and ten healthy controls who had ^18^F-THK523 studies. Conservative Z-scores greater than 1.5, indicating just 1.5 standard deviations (SDs) from the mean of the control participants, were considered abnormal.

## Results

### Demographic information

The demographics of the patients whose postmortem human brain tissue was utilized for these studies, expressed as mean ± SD, are presented in Table 1. All participants assessed were of similar age with comparable postmortem intervals for tissue collection. The patient with PSP who underwent PET was 79 years old and had 11 years of formal education. A neuropsychological examination revealed the patient had a Mini Mental State Examination score of 26, a Clinical Dementia Rating Scale score of 1 a Clinical Dementia Rating Scale–Sum of Boxes score of 5.5, an episodic memory composite score of -3.22 and a nonmemory composite score of -3.40. The PSP participant died 5 months after the PET scans were taken.

**Table 1:** **Patient demographics**^**a**^

**Diagnosis**	**No. of patients**	**Mean age (SD), years**	**Mean PMI (hours)**
AD	3	72.9 ± 6.7	22.8 ± 8.8
PSP	3	73.2 ± 4.6	37.2 ± 18.6
PiD	2	75.4 ± 7.1	47.0 ± 5.2
CBD	1	72.5	11.0
PD	1	70.5	22.5

^a^AD, Alzheimer’s disease; CBD, Corticobasal degeneration; PD, Parkinson’s disease; PiD, Pick’s disease; PMI, Postmortem interval; PSP, Progressive supranuclear palsy.

### Assessment of THK523 binding/fluorescence in non–Alzheimer’s disease tauopathies

**Figure 1:**
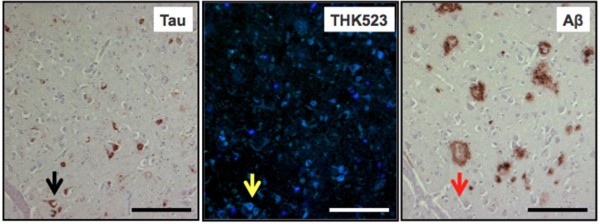
**THK523 binds to neurofibrillary tangles in an Alzheimer’s disease patient.** Microscopy of 5-μm serial sections from the hippocampus of a representative Alzheimer’s disease patient. The left image (tau) is the first of three serial sections. It is immunostained with tau polyclonal antibody to detect tau lesions in the hippocampus. The black arrow indicates the positioning of neurofibrillary tangles (NFTs). The positioning of the NFTs indicated by the black arrow was transferred to subsequent adjacent serial sections that were either stained with THK523 (middle image, THK523) or immunostained with a monoclonal antibody raised to amyloid-β (Aβ) to identify senile plaques in the tissue section (right image, Aβ). Fluorescence staining of THK523 (middle) appears to colocalise and resemble tau NFTs, indicated by the yellow arrow, in the absence of Aβ immunoreactivity in the same tissue region (right, red arrow). Tissue section images were obtained using a Zeiss microscope and an AxioCam digital camera (Carl Zeiss Microscopy, North Ryde, Australia). Scale bars, 100 μm.

**Figure 2:**
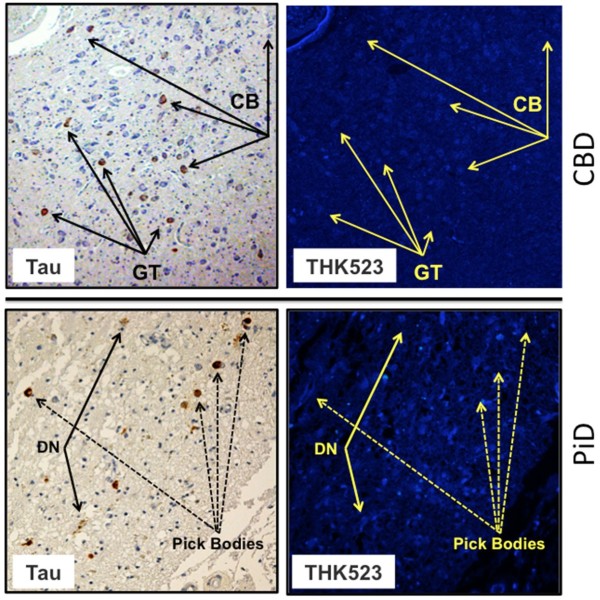
**THK523 does not bind to tau lesions in corticobasal degeneration or Pick’s disease.** Microscopy of 5-μm serial sections from the striatum of a corticobasal degeneration (CBD) patient (top panel) and the frontal cortex of a Pick’s disease (PiD) patient (bottom panel). The left side (Tau) shows the first of two serial sections immunostained with tau polyclonal antibodies to detect tau lesions. The arrows indicate the positioning of brown immunostained globose tangles (GT) and coiled bodies (CB; top panel) in a CBD patient and Pick’s bodies and dystrophic neurites (DN) (bottom panel). The same region of tissue was subsequently imaged for the adjacent section, which was stained with THK523 (right, THK523). The positioning of the tau lesion arrows was transferred to the adjacent stained serial section and is indicated by yellow arrows (THK523). The absence of fluorescence suggests that THK523 does not bind to CBD or PiD tau lesions. Tissue sections were imaged using a Zeiss microscope and an AxioCam digital camera at 5× (CBD) and 20× (PiD) original magnification.

**Figure 3:**
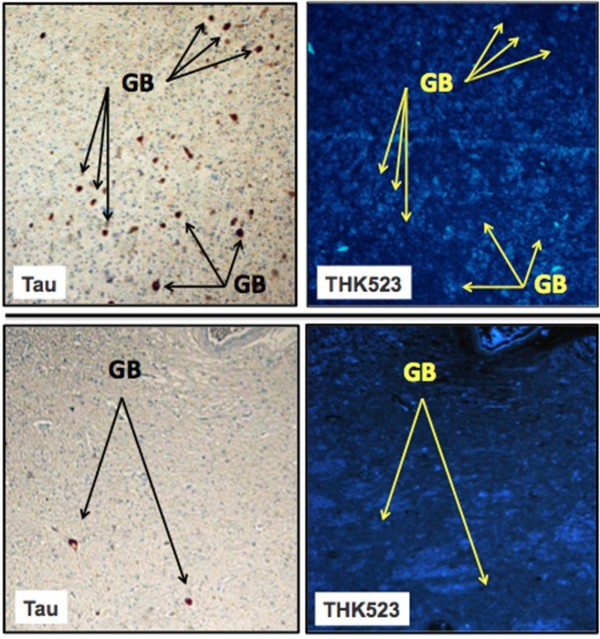
**THK523 does not bind to globose tangles in a progressive supranuclear palsy patient.** Microscopy of 5-μm serial sections taken from the pons (top panels) and the striatum (bottom panels) of a representative progressive supranuclear palsy (PSP) patient. Left (Tau) images show the first of two serial sections immunostained with a tau polyclonal antibody to detect globose tangles (GB). The black arrows indicate the positioning of brown immunostained GBs in the tissue section examined. The same region of tissue was subsequently imaged for the adjacent section, which was treated with THK523 (right, THK523). The positioning of the tau lesion black arrows was transferred to the adjacent stained serial section and is indicated by the yellow arrows (THK523). The absence of fluorescence suggests that THK523 did not bind to the tau lesions of the PSP patients examined. Tissue sections were imaged using a Zeiss microscope and an AxioCam digital camera at 5× original magnification.

**Figure 4:**
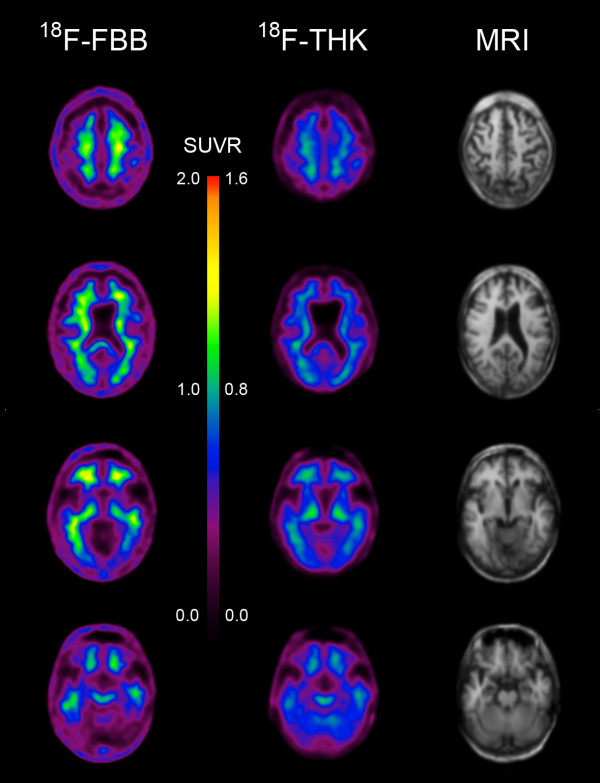
^**18**^**F-THK523 and **^**118**^**F-florbetaben positron emission tomography scans in a progressive supranuclear palsy patient.** Representative ^18^F-florbetaben (^18^F-FBB, left) and ^18^F-THK523 (^18^F-THK, right) transaxial images at three different brain levels of a 79–year-old PSP patient with a Mini Mental State Examination score of 26. Visual inspection reveals no cortical retention of either ^18^F-THK523 or ^18^F-florbetaben, despite a postmortem immunohistological examination (see Figure 3 5 months after PET evaluation), confirming the presence of tau lesions. SUVR, Standardised uptake value ratio.

The results of our previous postmortem studies [29] have indicated that THK523 labels AD tau lesions, namely, NFTs in the hippocampus of patients with AD (Figure 1). To determine whether THK523 would also bind to non-AD tau lesions, brain sections from non-AD tauopathies were evaluated. For these studies, fixed contiguous serial sections from the striatum (CBD and PSP samples), the frontal cortex (PiD sample) and the pons (PSP sample) were either immunostained with a polyclonal tau antibody for the detection of tau lesions or incubated with the fluorescent compound THK523 to determine whether THK523 bound to non-AD tauopathy aggregates. Immunohistochemical staining of brain tissue regions rich in tau immunoreactivity was detected by light microscopy, and the same tissue region within the adjacent serial section was assessed by fluorescence microscopy to compare and determine whether the immunoreactive tau lesion colocalised with THK523 binding, indicated by a fluorescent signal. Immunohistological assessment of brain sections from CBD and PiD patients revealed the characteristic presence of globose tangles, coiled bodies (as indicated by the arrowheads in the figures; see top left panel of Figure 2) and Pick’s bodies (arrowheads in bottom left panel of Figure 2) in the striatum and frontal cortex. Nonetheless, examination of the same region within the adjacent serial section exhibited no signs of THK523 fluorescence, indicating that THK523 does not bind to these tau lesions. Likewise, immunohistological evaluation of the pons (left, top panel of Figure 3) and striatum (left, bottom panel of Figure 2) in PSP patients revealed that, despite the presence of tau globose tangles, there was no detectable THK523 fluorescence signal in the same region of the adjacent serial brain section, again suggesting that THK523 does not bind to globose tangles. It is noteworthy that one of the three PSP patients evaluated (Figure 3) had had ^18^F-THK523 and ^18^F-florbetaben PET scans 5 months prior to death. There was low ^18^F-florbetaben cortical retention (Figure 4), correlating with postmortem results showing absence of Aβ deposits. There was also low cortical ^18^F-THK523 retention as well as low ^18^F-THK523 retention in the basal ganglia, midbrain, pons and cerebellar white matter (Figure 4), which was indistinguishable from age-matched controls and in contrast to the relatively high density of tau lesions observed in the brain, confirming the absence of THK523 binding to globose tangles. Analysis of the PSP patient PET scans showed global and regional Z-scores less than 1.0 for ^18^F-THK523 and ^18^F-florbetaben, confirming the visual inspection of the images.

### Assessment of THK523 binding/fluorescence in Parkinson’s disease

**Figure 5:**
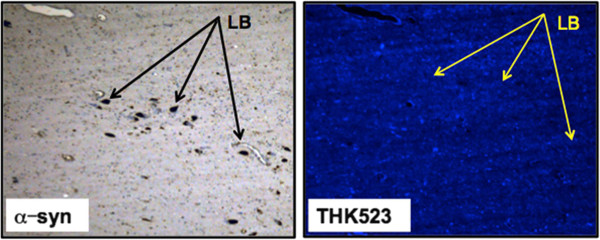
**THK523 does not bind to Lewy bodies in Parkinson’s disease patient.** Microscopy of 5-μm serial sections from the substantia nigra of a Parkinson’s disease patient. The left image (α-syn shows the first of two serial sections. It immunostained with an α-synuclein antibody to detect Lewy bodies (LB) in the substantia nigra. The black arrows indicate the positioning of LBs. The same region of tissue was subsequently imaged for the adjacent sections, which were treated with THK523. The positions indicated by the black arrows in the left panel were transferred to the adjacent THK523 serial section and are indicated by the yellow arrows (THK523). The absence of fluorescence staining indicates that THK523 does not bind to α-synuclein containing Lewy bodies in the same tissue region. Tissue sections were imaged using a Zeiss microscope and an AxioCam digital camera at 5× original magnification.

To further test the selectivity of THK523, we evaluated its ability to bind to Lewy bodies composed of α-synuclein and ubiquitin aggregates sharing a similar β-sheet secondary structure. For these studies, serial sections of the substantia nigra from a PD patient were either immunostained with antibodies raised to α-synuclein, or treated with a fluorescent compound, THK523. Evaluation of these stained serial sections demonstrated that, whilst the presence of Lewy bodies could be clearly identified by immunohistochemistry (Figure 5, left panel), the adjacent serial section was devoid of THK523 fluorescence (Figure 5, right panel), implying that THK523 did not bind to Lewy bodies.

## Discussion

In the present study, we further characterized ^18^F-THK523 as a selective tau imaging agent by testing its ability to recognize the various morphological conformations of tau in a wide spectrum of tauopathies. Whilst in our previous studies we determined that THK523 binds selectively to NFTs in preference to Aβ plaques [29,32], in this study we also assessed ^18^F-THK523 binding to other β-sheet structured protein fibrils, namely, α-synuclein-containing Lewy bodies.

Given the morphological and ultrastructural diversity of tau aggregates, it may be unlikely that a single tau imaging agent could be useful for the diagnosis of all tauopathies. In the first instance, tau comprises six isoforms distinguished by their length and number of repeats (R) of microtubule binding domains [6,42]. AD tau comprises an equal ratio of the 3R and 4R isoforms, which mainly appear as NFTs. The 4R isoform predominates in PSP with tau aggregates comprising tufted-shaped astrocytes, GTs and oligodendroglial coiled bodies [43,44]. Despite also being a 4R tauopathy, in CBD the tau inclusions appear as astrocytic plaques, neutropil threads and tau pretangles [45]. PiD, a 3R tauopathy, is diagnosed by the presence of ‘Pick bodies’, tau-positive intraneuronal inclusions [46]. Moreover, these tau aggregates are further differentiated by their ultrastructure. NFTs are predominantly composed of paired helical filaments (PHFs), tau inclusions in PSP and CBD are composed predominantly of straight tau filaments (SFs) and twisted tau filaments (TFs) [11], whereas Pick bodies comprise a combination of TFs and random coiled tau filaments [11]. It is noteworthy that, whilst PSP and CBD share SFs, the size of the filaments is significantly different [47]. Despite this diversity, a recent report describing a novel class of tau tracers phenyl/pyridinyl-butadienyl-benzothiazoles/benzothiazoliums (PBB) demonstrated binding to a variety of tau deposits in fluorescence studies of AD, CBD and PSP brain sections [48]. Additionally, that study also demonstrated positive [^11^C]PBB3 PET scans in both AD and CBD patients [48].

Given the evident differences in THK523 staining, the fluorescence microscopy studies we present herein demonstrate that THK523, even at the very high concentration of 100 μM, does not bind to non-PHF-tau aggregates. The existence of a THK523 binding site on PHFs that is absent in the other conformations is further emphasized by previous computerized cross-sectional and fragmentation studies which indicated that, whilst these types of filaments share a similarly shaped morphological unit, the filament arrangement is different [49].

PHFs appear as two filaments twisted around one another with a cross-over repeat of 80 nm and an apparent width varying between about 10 nm and 22 nm [50]. The resulting aggregate exhibits an amyloid structure characterized by a β-sheet network forming the heart of the protofibril network. This ultrastructural property shared with Aβ and α-synuclein aggregates sometimes results in the nonselective binding of imaging agents [51]. As noted previously [29], in addition to THK523’s binding to NFTs, our fluorescence studies obtained at high tracer concentrations—10,000-fold higher than the concentrations typically achieved during a PET scan—demonstrated inconsistent THK523 staining of Aβ plaques. THK523 stained the dense core of some Aβ plaques in the frontal cortex of AD sections but did not stain dense Aβ plaques in the hippocampus (Figure 1, right panel). It is noteworthy that variable staining of NFTs at high concentrations of PiB has also been reported [52]. In addition to previous reports of *in vitro* studies [29,30], several lines of evidence support the notion that THK523 selectively binds to PHF-tau and does not bind to Aβ *in vivo*: (1) Cortical THK523 retention is significantly higher in AD; (2) THK523 retention follows the known distribution of PHF-tau in the AD brain; (3) PiB and THK523 show different brain regional distribution patterns; (4) hippocampal THK523 retention significantly correlated with cognitive parameters, but hippocampal PiB retention did not; and (5) hippocampal THK523 retention significantly correlated with hippocampal volume, but hippocampal PiB retention did not [32].

The selectivity of THK523 for tau over other β-sheet aggregated proteins was further demonstrated by fluorescence microscopy studies showing the absence of THK523 fluorescence in brain sections exhibiting immunolabelled α-synuclein-containing Lewy bodies (Figure 5, right panel).

The PSP patient showed neither ^18^F-THK523 nor ^18^F-florbetaben retention in the brain, suggesting the absence not only of Aβ plaques but also of tau deposits. Neuropathological examination of the brain confirmed the absence of Aβ plaques; however, typical tau lesions were present in different brain regions that were not stained by THK523. Given the ultrastructural diversity of tau aggregates, the information derived from these THK523 studies is highly valuable for the future design of tau imaging ligands.

## Conclusion

In the present study, we have demonstrated that THK523 selectively binds to PHF-tau with negligible binding to PSP, CBD and PiD tau aggregates, as well as to Aβ and α-synuclein aggregates. The results of this study also show that novel tracers that bind to non-PHF tau aggregates are needed.

## Abbreviations

AD: Alzheimer’s disease; Aβ: Amyloid-β; CBD: Corticobasal degeneration; CDR: Clinical Dementia Rating Scale; CDR-SOB: Clinical Dementia Rating Scale–Sum of Boxes; CSF: Cerebrospinal fluid; FTLD: Frontotemporal lobar degeneration; GM: Grey matter; MMSE: Mini Mental State Examination; NFT: Neurofibrillary tangle; PET: Positron emission tomography; PiB: Pittsburgh compound B; PiD: Pick’s disease; PSP: Progressive supranuclear palsy; ROI: Region of interest; SF: Straight filament; SUV: Standardised uptake value; TF: Twisted filament.

## Competing interests

The authors declare that they have no competing interests.

## Authors’ contributions

VLV, MTF-T, KY, NO and CLM designed the experiments. SF, RSM, RH, KY, YK and NO designed and manufactured THK523. LT and IB planned and conducted the human brain section immunostaining experiments. CAM planned and conducted the pathological characterisation of human brain samples. VL and CCR planned and coordinated human PET studies. MTF-T and VLV drafted the manuscript. All authors read and approved the final manuscript.
